# Thirty-Fifth Annual Session of the New Jersey State Dental Society

**Published:** 1905-09

**Authors:** William H. Trueman


### THIRTY-FIFTH ANNUAL SESSION OF THE NEW JERSEY STATE DENTAL SOCIETY.

THE thirty-fifth annual session of the New Jersey State Dental Society convened at the Auditorium, Asbury Park, Wednesday morning, July 19, 1905.

After the usual preliminaries of opening the session, the President, Dr. W. G. Chase, M.D., D.D.S., of Princeton, read his address. This was brief, and referred to matters of local interest only. The society then, after considering various business matters, adjourned until evening.

At the evening session, Dr. Sinclair Tousey, of New York, read a paper upon "The X-Ray and High Frequency Currents in Dentistry," illustrating by lantern slides the various applications of the X-Ray in dental diagnosis, and a number of instruments to facilitate its use in dental radiography. After reading his paper, the doctor demonstrated his dental fluoroscope, an instrument that is practically a mouth mirror and fluoroscope combined. While it does not reveal to the observer minute detail as does a successful radiograph, it will, in many cases, show all that is required at a glance, and render unnecessary the labor and technical skill involved in developing a photographic film. At present, the required apparatus is somewhat cumbersome; it is expensive, and requires for its proper use a measure of technical skill all dentists do not possess. It is not likely that it will long so remain. We may confidently look for this new aid in accurate diagnosis to become, in the near future, so simplified as to be available to every progressive dentist. Dr. Tousey's dental fluoroscope is a step in that direction.

MORNING SESSION, JULY 20.

Dr. Robert H. M. Dawbarn, of New York, was on the programme to read a paper upon "Adenoids." He had his paper with him, but preferred to lay it aside and make his remarks extempore. Fluently, and in a very interesting manner, he proceeded to give a history of the step by step unravelling of the mystery surrounding and the baneful effects attributed to these growths since they were first recognized some thirty-six years ago. No abstract can do justice to his clear and instructive explanation of their origin, the anatomical peculiarities of the parts near which they are found, and the reasons why their presence as an irritant, as an obstruction, or as an aggravation of diseased or abnormal conditions is so marked and far reaching. He minutely described how they are diagnosed, and laid especial stress upon the more noticeable signs which indicate their presence. Inability of a very young infant to continuously nurse usually indicates the presence of adenoids; the nasal passages being partly closed, the infant is compelled, after a few efforts, to stop to breathe. Later in life may be noticed mouth-breathing, a peculiar facial expression, and changes their presence brings about in the maxilla, the relation of the maxilla to the mandible, and in the arrangement of the teeth. A change in the voice, and more serious and far-reaching disturbances, mental and physical, due to their interference with respiration, are noted later.

He spoke at length of the general prevalence of adenoids in cities on the coast-line, suggesting that this was due to climatic conditions, to frequent and sudden marked changes in humidity and temperature. Some children seem to be afflicted with them almost from birth; he had removed them from infants of but a few months. He advised their prompt removal as soon as discovered, under a general anæsthetic, preferring for this purpose "ether." In the case of very young children, he inquired of the mother when the child took the usual mid-day nap, and selected that time for the operation, operating at the child's home. On arriving, he cautiously, and noiselessly, approached the sleeping infant, and, with care not to disturb it, administered a minute dose of chloroform. This acts quickly, the little patient passing quietly from normal sleep to partial anæsthesia without being aroused, —inhaling a few drops is frequently sufficient. This is followed by ether until the full anæsthetic stage is reached. The operation is then performed, and after it is over the infant is returned to its couch to find its nap. On awakening, it scarcely knows that anything unusual has taken place. Careful attention to diet for a few days, and the little one's trouble is over. By this procedure the child is saved the fright from which it otherwise would have suffered before the operation, and the reaction following this excitement. For this the mother is duly thankful.

He was not favorably impressed with the use of cocaine in this operation, preferring a general anæsthetic. He directed attention to the profuse hemorrhage frequently attending the removal of adenoids, and to the care necessary to prevent this becoming a serious complication. This he controlled by the application of very hot water. He dipped a napkin in boiling water, and after lightly wringing out the excess of water immediately applied it to the parts. The patient, being under an anæsthetic, felt no pain; it acted promptly, and he had not in any case seen any but beneficial results follow its use. It had been suggested that the application of water so hot would be followed by sloughing; this he had never seen.

He urged the importance of prompt action in all cases of adenoids; they never get better of themselves, and it is very important that they should be removed before they have caused structural changes that will be permanent, or have set up a diseased condition difficult to control, or it may be incurable.

He next referred to the tonsils, which frequently are involved, or are a complication in cases of adenoids. They may be the seat of operative procedure as the result of adenoids, in connection with them from causes not due to their presence, or on account of abnormal or diseased conditions peculiar to the tonsils themselves, and in cases where adenoids are not found. He described minutely the anatomy of the tonsils and their surroundings, pointing out the special features which make these glands so liable to give trouble, and which so greatly favor microbe invasion. He made the broad distinction between operations for the removal of adenoids and operations upon the tonsils. While the first rarely, if ever, results fatally, the latter is much more serious, and has repeatedly compromised life. This he urged as another reason for the prompt and thorough removal of adenoids, lest its neglect should make necessary the more serious and more dangerous operation. He objected to the use of cocaine when the tonsils are to be extirpated on account of its causing contraction of the tissues, which may lead to some portion of the organ being overlooked, and necessitate a second operation. If a local anæsthetic is used he preferred beta-cocaine, which, while equally efficient as an anæsthetic, is not open to this objection. Hemorrhage, in operation upon the tonsils, is frequently dangerously profuse. The danger is not alone from blood flowing into the air-passages, etc. The abstraction of blood, especially if the patient be anæmic, he considered more dangerous to life than the so-called surgical shock, especially when it comes from, and lessens the normal blood-pressure in vessels concerned in the proper nourishment of the brain. This hemorrhage can be controlled, and in many cases may be absolutely prevented by first ligating the gland in a manner he described and illustrated, but which cannot be explained without a diagram.

The subject presented he considered proper for discussion by a dental society. It is one of much importance to the dentist, and to the stomatologist, not solely because it concerns territory adjacent to that especially under dental care, but rather because the dentist far more frequently and more carefully examines this field than does the medical practitioner. His opportunity and his special training enables him to quickly detect abnormal conditions in and about the oral cavity, and whether or not he undertakes their treatment, he should be able to appreciate their character and importance. By announcing the presence of such as require prompt surgical interference, he not only renders his patient an excellent service, but advanced his own reputation with the community, and the standing and importance of the profession he honors.

The discussion added but little of importance; it, however, enabled the doctor to elucidate some points upon which further information was desired, and to know that his remarks were fully appreciated.

THURSDAY EVENING, JULY 20.

Dr. A. W. Harlan, of New York, read a paper entitled "Food in its Relation to Teeth—Their Sockets and Adjacent Structures."

The paper was somewhat lengthy. It brought up to date matters which have been in the past frequently discussed. The essayist made free and discriminating use of information derived from experiments recently made at Washington, to determine by actual tests under exacting scientific control the value of various foods, the effects of food preservatives upon the human economy, and the relation of diet to nutrition.

To counteract the probable effect of reading a somewhat prosy paper, the doctor ingeniously followed it by a few humorous lantern slides of subjects presumed to be related to that of his paper. It was well done. The slides and his witty remarks were well appreciated.

The discussion was along lines that developed nothing especially new. The paper, indeed, was not one that could be profitably discussed by those who had not made of the subject a careful and continued study. It will read well, and will be instructive.

This was followed by the reading of a paper upon "Sensitive Dentine," prepared by Dr. C. S. Stockton, who, on account of recent illness, was unable to be present. It detailed a method the doctor has recently been using with satisfaction for obtunding sensitive dentine, in which dryness and warm air seemed to play an important part.

These four papers completed this portion of the programme.

Of the fifty-three clinics on the programme, the writer saw but few. Probably the most novel, and the most instructive, was a collection of anatomical sections of the head and face from the museum of the Dental Department of the University of Pennsylvania, presided over by Dr. F. A. Faught, of Philadelphia. These were made by Dr. Cryer during his study of the anatomy of the head and face with special reference to the various cavities which so frequently become the field of surgical operations. The human head has been "sliced" into parallel sections showing, graphically, the exact relation, at all points, of its various parts as could be done in no other way. This instructive exhibit was made much more so by the lucid explanations Dr. Faught was able to give of its purpose, and of the various points of dental interest invisible in an undivided skull, many of which are but imperfectly shown in anatomical plates. Indeed, until Dr. Cryer devised this method of investigation they were but imperfectly known.

The manufacturers' exhibits were not equal to the best of former years. This was due, no doubt, to the extra effort to display their goods made so recently at the meeting of the Pennsylvania State Dental Society, in Philadelphia. They were, however, numerous and instructive, and proved a convenience to those living at a distance from the large cities.

The Ransom & Randolph Company, of Toledo, Ohio, had a new anæsthetic apparatus by which it is said prolonged anæsthesia can be maintained without loss of consciousness. This is said to have been in use, experimentally, for several years, and to be safe and satisfactory. It is known as "Weisell's Airoform and Airoform Appliance." The Airoform is a proprietary preparation consisting of chloroform, cologne spirit, amyl nitrite, and oleum unonæ. The apparatus is so constructed that the exact proportion of the anæsthetic and atmospheric air passing through it can be determined; this is so regulated that sufficient only of the anæsthetic is used to produce the desired effect,—insensibility to pain without loss of consciousness.

Dr. Brinkman, Chairman of the Clinic Committee, has overcome the difficulty so frequently encountered, of providing chairs for operative clinics. An ordinary steamer chair is fitted with a head-rest. A box to contain it when folded for packing, is furnished on one side with cleats to hold the chair in position, and serves as a platform on which the chair stands when in use. This box will hold not only the chair, but also a folding-table, a basin, a water-pitcher, and a cuspidor—a complete outfit. When in use, the chair, table, cuspidor, basin, and water-pitcher provide each operator with a very serviceable and convenient suit of furniture that, with a white paper table-cover, looks neat and inviting. The outfit is quite inexpensive, and packs not only into a small compas, but into a form that is readily and safely stored with the least possible loss of room, and when needed is quickly unpacked, and easily handled. The society has provided a number of these, and has thus happily solved the chair question. They were used for the first time at this session, and well filled the bill.

William H. Trueman.

